# Multi Ceramic Particles Inclusion in the Aluminium Matrix and Wear Characterization through Experimental and Response Surface-Artificial Neural Networks

**DOI:** 10.3390/ma14112895

**Published:** 2021-05-28

**Authors:** Ballupete Nagaraju Sharath, Channarayapattana Venkataramaiah Venkatesh, Asif Afzal, Navid Aslfattahi, Abdul Aabid, Muneer Baig, Bahaa Saleh

**Affiliations:** 1 Department of Mechanical Engineering, Malnad College of Engineering, Hassan, Affiliated to Visvesvaraya Technological University, Belagavi 573201, India; cv@mcehassan.ac.in; 2 Department of Mechanical Engineering, P. A. College of Engineering, Affiliated to Visvesvaraya Technological University, Belagavi, Mangaluru 574153, India; 3 Department of Mechanical Engineering, Faculty of Engineering, University of Malaya, Kuala Lumpur 50603, Malaysia; navid.fth87@yahoo.com; 4 Engineering Management Department, College of Engineering, Prince Sultan University, P.O. Box 66833, Riyadh 11586, Saudi Arabia; aaabid@psu.edu.sa (A.A.); mbaig@psu.edu.sa (M.B.); 5 Mechanical Engineering Department, College of Engineering, Taif University, P.O. Box 11099, Taif 21944, Saudi Arabia; b.saleh@tu.edu.sa

**Keywords:** B_4_C, Gr, Al2219, delamination wear, MML, MMCs, artificial neural networks

## Abstract

Lightweight composite materials have recently been recognized as appropriate materials have been adopted in many industrial applications because of their versatility. The present research recognizes the inclusion of ceramics such as Gr and B_4_C in manufacturing AMMCs through stir casting. Prepared composites were tested for hardness and wear behaviour. The tests’ findings revealed that the reinforced matrix was harder (60%) than the un-reinforced alloy because of the increased ceramic phase. The rising content of B_4_C and Gr particles led to continuous improvements in wear resistance. The microstructure and worn surface were observed through SEM (Scanning electron microscope) and revealed the formation of mechanically mixed layers of both B_4_C and Gr, which served as the effective insulation surface and protected the test sample surface from the steel disc. With the rise in the content of B_4_C and Gr, the weight loss declined, and significant wear resistance was achieved at 15 wt.% B_4_C and 10 wt.% Gr. A response surface analysis for the weight loss was carried out to obtain the optimal objective function. Artificial neural network methodology was adopted to identify the significance of the experimental results and the importance of the wear parameters. The error between the experimental and ANN results was found to be within 1%.

## 1. Introduction

Metal matrix composites (MMCs) have gained much interest recently. They have been used extensively in aircraft, space shuttles, automotive, commercial airliners, electrical substrates, bicycles, and motorcycles, as well as in a wide range of other applications [1,2]. Recently, numerous automotive makers worldwide have been making great strides to use aluminium metal matrix composites (AMMCs) in disc brakes instead of traditional cast iron [3,4]. Aluminium (Al) matrix strengthened with hard ceramic particles have arisen as a promising material for wear-resistant and lightweight applications [5]. The inclusion of strong ceramic particles into the Al-matrix contributes to a substantial improvement in the material’s hardness [6]. For structural purposes, ceramic materials are appropriate and have been used for a variety of tribological applications, particularly over the past two decades [7]. It is universally believed that increasing the Al matrix’s wear resistance is done by increasing the ceramic particle content [8]. The inclusion of silicon carbide (SiC), aluminium oxide (Al_2_O_3_), boron carbide (B_4_C), TiB_2_, and MoS_2_ in the Al matrix can have a detrimental effect on the composites’ wear properties [9].

Surappa et al. (1982) reported that Al-5 wt.% Al_2_O_3_ exhibited better abrasive wear resistance than Al reinforced with 8 wt.% Si and 8 wt.% Si. The findings show that Al_2_O_3_ could be used as a wear-resistant and abrasion-resistant and could be a replacement for silicon in Al [10]. Liang et al. (1995) studied the inclusion of SiC in the Al matrix, which resulted in increasing the surface hardness and wear resistance of the composite. The powder metallurgy method was used to produce 15 vol.% SiC composite particles 3.5, 10, and 20 µm in size. The particle size has a substantial impact on the wear behaviour of particulate-reinforced Al composites [11]. Krakhmalev et al. (2006) carried out a spark plasma sintering process to prepare the Mo–Si–Al composite, which resulted in reduced abrasive wear, affecting the composite’s wear resistance through the peel up of Al_2_O_3_, and the tribo-chemical layer that formed led to a low wear resistance [12]. Szu Ying Yu et al. (1997) studied two varieties of MMCs fabricated using the hot extrusion process. SiC whisker and SiC particulate were chosen to strengthen the Al matrix. The wear resistance of Al 6061 was improved for all the temperature ranges of 25–300 °C when strengthened by SiC particles [13]. M. Kok et al. (2005) reported a high percentage of porosity when fine Al_2_O_3_ was used as a reinforcement through the vortex method. In this study, the particle’s size and weight increased the density of the composites [14]. Doel et al. (1996) studied Al–SiC composites and concluded that the inclusion of SiC led to the increased tensile strength of the matrix [15].

Mohammad Sharifi et al. (2011) noticed that SiC and B_4_C were the most widely used ceramic reinforcing materials for Al alloys. In this study, growing the amount of B_4_C nanoparticles in the matrix resulted in increased hardness; the 15 wt.% B_4_C reinforced composite possessed the highest hardness of 164 HV [16]. Feng Tang et al. (2008) produced Al5083–B_4_C composites using powder cryo-milling, and the Al5083-10% B_4_C composite showed better wear resistance (40%) when compared with other combinations [17]. Alizadeh et al. (2015) studied the wear and creep behaviour of MMCs. Al-based composites were produced through the powder metallurgical approach and the strengthening of B_4_C and carbon nanotubes (CNTs) inclusions was focused on. The presence of B_4_C particles in the Al matrix improved the resistance to creep and wear, whereas 10% B_4_C showed a greater creep and wear resistance [18]. Saraswat et al. (2018) prepared MMCs using pure Al as a matrix, strengthened by B_4_C through a stir casting process. The hardness and wear resistance of the Al matrix was improved because of the presence of B_4_C [19]. Shorowordi et al. (2003) noticed the distribution of B_4_C particles in the matrix was good compared with the distribution of SiC and Al_2_O_3_ in the matrix. B_4_C is an appealing reinforcing material for all ceramic materials because of its exceptional chemical and thermal stability [20]. Ipek (2005) selected Al4147 and 85–130 µm B_4_C and SiC particles to prepare composites through liquid casting. The inclusion of B_4_C particles in the Al matrix increased the composite hardness by 41% [21].

Auradi et al. (2014) stated appreciable improvements in the ultimate tensile strength (UTS = 44.35%) because of the incorporation of B_4_C in Al6061. The K_2_TiF_6_ + B_4_Cp mixture ensured better wettability, so that an appreciable distribution could be achieved [22]. Kala et al. (2014) observed that the existence of B_4_C and SiC in the matrix led to an increase in UTS with a decrease in hardness and ductility. Gr (graphite) inclusion in the aluminium matrix helped to reduce the friction coefficient. While hybrid ceramic reinforcement has greatly improved the mechanical properties, there is a dearth of evidence on the tribological properties of hybrid reinforcement [23]. Cho et al. (2006) described the impact of three separate solid lubricants in a brake friction material (Gr, Sb_2_S_3_, and MoS_2_) on various aspects of friction characteristics. According to the findings reported, Sb_2_S_3_ and Gr ensured friction stability and decreased resistance. At a high temperature, Gr altered the chemical compositions and showed various tribological performances [24]. Suresha et al. (2010) stated that Gr enhancements were revealed to be helpful in reducing wear in Al matrix composites because of their solid lubricant property. Gr usage in the composites was limited, to a certain extent, above which its inclusion for strengthening was not advantageous [25]. Hu et al. (2000) studied fatigue and fretting wear. Further notable fatigue and fretting wear increased with 0.4% carbon steel if 20 vol.% of SiC particles were added to the Al2124. The liners were constructed from an Al alloy strengthened with graphite and alumina and were used to build the engine block [26]. LLorca et al. (2002) mentioned more benefits offered by Al reinforced with discontinuous particles, including wear, which increased the thermal related properties of the matrix [27]. Amin Bahrami et al. (2015) studied the properties of (Al-7 wt.% 10Ce-TZP/Al_2_A_3_) nanocomposites. Friction and wear characterization were made at different sintering temperatures (400 °C, 450 °C, and 500 °C); 450 °C was a significant temperature for sintering during the fabrication (powder metallurgy) of the nanocomposite. It was found that the base alloy hardness and wear resistance were greatly improved by the contributions of ceramic particles [28]. Amin Bahrami et al. (2018) mentioned that the reaction products of Al regulated the wettability of B_4_C substrates with B_4_C particles [29]. Amin Bahrami et al. (2016) described the contribution of each of the parameters on the bending strength of the resulting bilayer composites and indicated that the parameter with the most significant effect was process temperature, with a contribution of 79%. Thermodynamic analysis and a thorough study of the microstructure allowed for establishing conditions for diminishing the effect of the Al_4_C_3_ phase [30]. Rao et al. (1996) studied the properties of Al2219 and its suitability in various applications. This material was used in the design of liquid cryogenic fuels tanks, including fluid oxygen and liquid hydrogen [31]. Siddesh Kumar et al. (2014) noticed aerodynamic shaped components could be easily obtained with Al2219, and that it was easy for designers as well, as it had excellent weldability. Al2219 has been used with considerable results on numerous launch vehicles, including Apollo, Saturn V, and Space Shuttle. Hence, Al2219 has effectively been used in aerospace and elevated temperature applications [32]. Mohammed et al. (2009) mentioned that artificial neural networks (ANNs) were used as a tool for modelling artificial intelligence. ANN was used to examine the impact of specific parameters of the drilling process. It is inferred that ANN is an outstanding analytical model that can be used to analyze other processes [33]. Kumar et al. (2008) explored the scope for the neural network used in assessing the wear loss of an Al390 alloy and stated that ANN could be effectively used in the field of material characterization and tribology as a prediction technique. The weight loss of aluminium 390 (A390) was predicted by well-optimized and well-trained neural networks [34].

Very little data are available on the dry sliding wear characteristics of the multi ceramic reinforced composites. Despite these complexities, the present research set out to establish the impact of hybridized composites’ wear characteristics (Al2219 + B_4_C − Gr). Al2219 was used as the matrix, and B_4_C and Gr were used as primary and secondary reinforcements, respectively. To improve the veracity of judgment, the L27 orthogonal array (Taguchi model) was used to discern the dry sliding, wear characteristics of the hybridized composite, and the un-reinforced Al2219. The test samples were subjected to a hardness test and wear test to study the hardness and wear behavior, respectively. SEM and XRD techniques were used to evaluate the microstructure and phase analysis, respectively. The ANN approach was explored for its suitability in predicting the significance of experimental results and identifying the prominent factors on the hybrid composites’ wear characteristics.

## 2. Materials and Methods

Al2219 appears to be the most viable material for the manufacture of liquid cryogenic rocket fuel tanks, cylinder heads, and in the automotive sector. It has a rare combination of properties, such as high strength-to-weight ratios and cryogenic characteristics, including weldability. In addition, more and more modern maritime vessels are being constructed from Mg-based aluminium (3–6%), as these alloys are exceptional in fresh and seawater resistance to corrosion [31]. Therefore, Al2219 was chosen as the matrix material, and its chemical composition is shown in Table 1. Among the ceramics, B_4_C has excellent wettability with the Al matrix because of the formation of the B_2_O_3_ layer during solidification [35]. It has an incredibly high neutron absorption capacity and belongs to the lightweight (2.52 g/cm^3^) ceramic material group. One of the best approaches for stiffening AMMCs is by adding B_4_C [36]. Adding B_4_C alone in greater quantities was found to not be favourable [37]. Hence, secondary reinforcement was necessary. It was pointed out that the inclusion of solid lubricants (MoS_2_, Gr, and BN) in AMMCs increased the wear resistance of composites. Gr has an incredibly low friction coefficient because of its strong crystal structure [38]. Many researchers have noticed that switching to hybridization is a significant way to enhance the metal matrix composites’ properties. The main objectives of the present research were to further the wear properties and hardness of the Al–Cu–Mg based metal matrix composites by an appropriate combination of opted reinforcements. Because of the excellent properties of the ceramic combination used, it could influence the alloy to provide a better tribological performance, to some extent.

### 2.1. Fabrication of Composites by Using the Stir Casting Method

**Figure 1:**
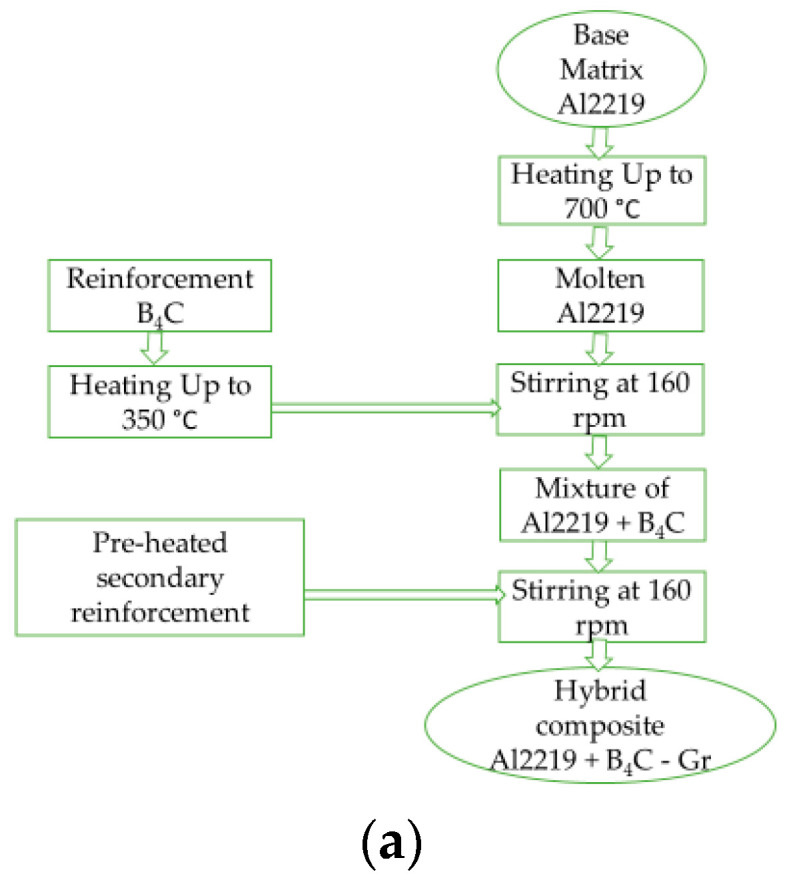

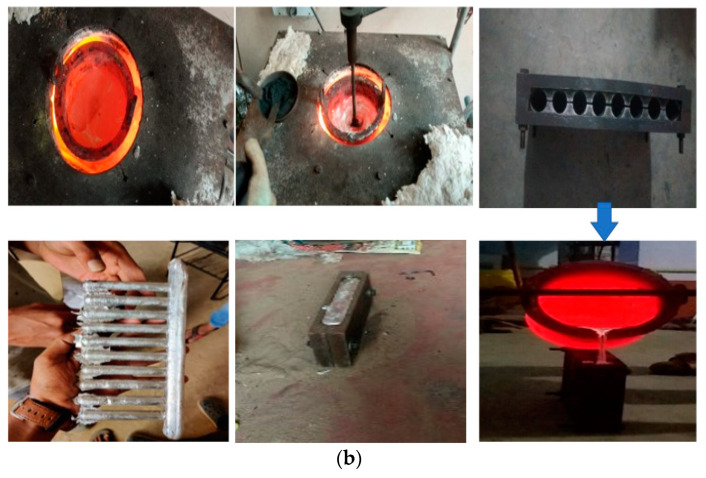
(**a**) Methodology adopted for hybrid composite casting: (**b**) Steps for hybrid composite casting.

While manufacturing MMCs, the major contention is the chemical compatibility between the matrix phase and the reinforcement, especially when using a liquid metal casting process, such as the stir casting process. Stir casting is adopted in the present research, as it is attractive and flexible and is relatively economical compared with other casting processes. This process is used to get homogeneous mixing of the reinforcement within the liquid matrix phase [39,40]. Here, graphite crucible was used for melting, in which the Al2219 alloy billet was held at 700 °C. Magnesium was added to the crucible to provide wettability. A zirconium coated steel stirrer was used to stir the molten mass at 160 rpm for about 5–10 min. The incorporation of primary reinforcement B_4_C accompanied this at a lower speed. Secondary reinforcement Gr was added to the premixed slurry, and stirring was continued for a further 5–10 min at 160 rpm to get proper mixing before pouring the molten slurry into the mould cavity. The molten slurry was inevitably poured into the graphite mould; then, it was allowed to solidify. Both the primary and the secondary reinforcements (Table 2) of particle size 30–50 µm were preheated at 350 °C. The steps involved in the casting are schematically represented in Figure 1a,b. The fabricated cast was later subjected to machining according to G99 standard (10 mm diameter and 30 mm length) in order to carry out the wear test.

### 2.2. X-ray Diffraction and Microstructural Study

Minor flaws can inflict severe damage to the stability of the material. The benefit of SEM is that a high degree of magnification and an exceptional depth of focus help visualize the particle distribution in the matrix material. Voltages in the range of 2 to 40 kVa and an electron beam of <0.01 μm in diameter were focused on the sample. X-ray diffraction tests were carried out with the Advanced Goniometer Model 2036E201 (Bruker: Bangalore, India) aid using Cu Kα radiation (Kα = 154,056). During the test, the sample was stagnant and scanned with a rate of 2 °/min through 10–80° for the diffraction angle (2θ) [41,42,43].

### 2.3. Hardness Test

The Brinell hardness samples were prepared according to the ASTM E10 standard (ISO 6506:2005) [44]. The hardness test (Model: PHB-3000, Indiamart, Bangalore, India) of the sample was conducted at a load of 250 kgf with a dwell time of 30 s.

### 2.4. Wear Test

The experimental runs were framed according to the design of experiments (L27-Taguchi model) approach. All the tests were performed using a pin on the disc (friction monitor TR-20-PHM 400, Make: DUCOM, Bangalore, India) set up attached with the data acquisition system. To prepare the wear test samples, ASTM G99 standard specifications were adopted [40]. The impact of the chosen parameters on weight loss was analyzed using an ANN (IBM-SPSS Statistics 22) framework [34]. The apparatus’s basic operating theory was to slide a pin against a disc (EN31 steel). The disc rotated at 1.25 m/s, 2.5 m/s, and 3.75 m/s and applied a load of 20 N, 30 N, and 40 N, with a sliding distance of 400 m, 600 m, and 800 m, respectively. These process parameters were used to describe the wear resistance of the AMMCs [40].

## 3. Results and Discussion

### 3.1. X-ray Analysis and Microstructural Study

**Figure 2:**
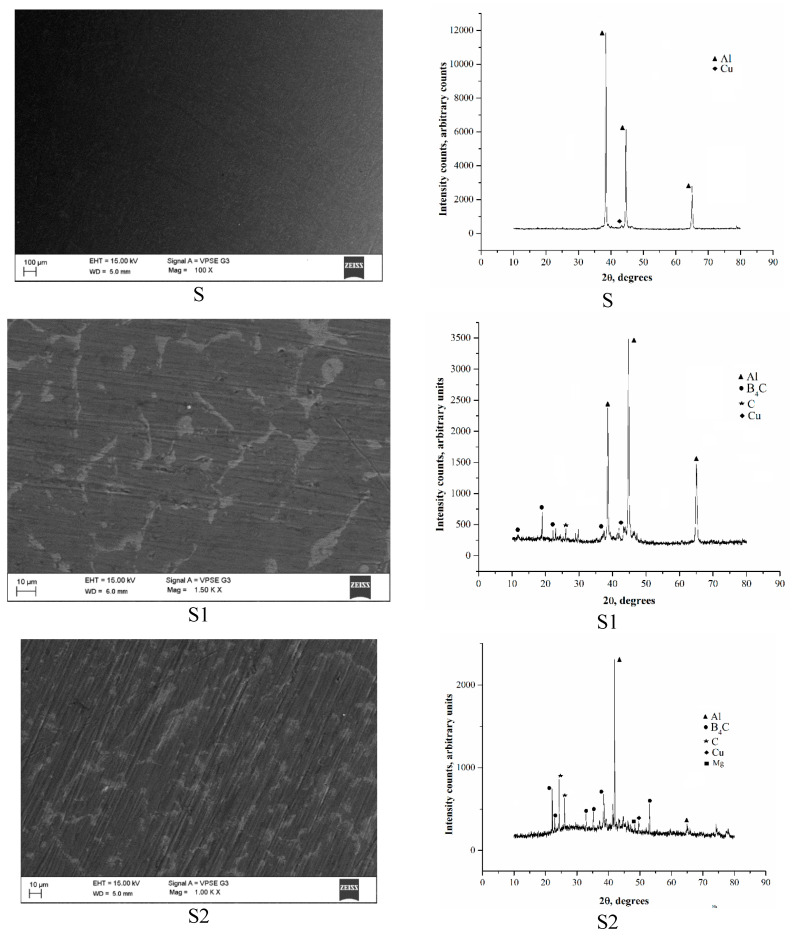

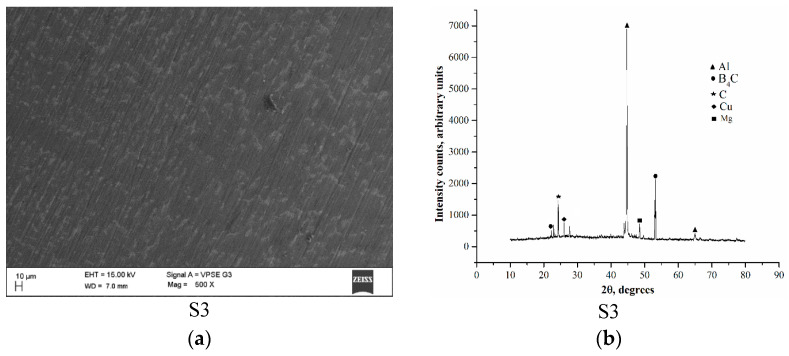
(**a**) SEM of Al2219, and aluminium metal matrix composites (AMMCs); (**b**) XRD of Al2219 and AMMCs.

SEM is essential for determining the damage caused by fractures. Microscopic studies have allowed for the detection of material defects and processing defects. There was no slag in the hybrid composite; equiaxed grains formed, as is clearly visible through SEM micrographs shown in Figure 2. Because of the mould surface and reinforcement particles’ presence, the degree of supercooling was reduced; therefore, many tiny nuclei formed during solidification. A large number of nucleation sites adjacent to the mould walls influenced the partial equi-axie grain formation, as shown in the hybridized samples. The cooling rate in conventional Al2219 was high compared with the hybrid composite. The nearest neighbouring gap between the nuclei was small, suppressing the crystal’s grain size, as seen in Samples S2 and S3. Reinforcement materials acted as nuclei agents, resulting in boundaries being formed, as seen in Samples S2 and S3. From Figure 2, it is observed that the samples had a low porosity and resulted in better interfacial bonding, helping to better distribute reinforced particles in the matrix.

**Figure 3:**
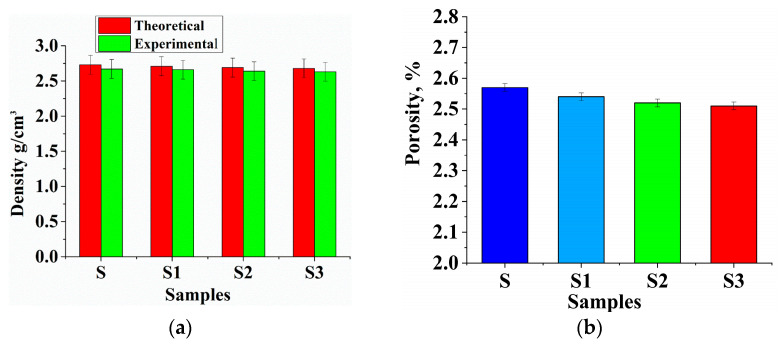
(**a**) Theoretical vs. experimental density of the samples; (**b**) sample’s porosity in %.

The XRD analysis confirmed the presence of Al with a higher peak. The presence of B_4_C and Gr was evident from smaller peaks in the S1, S2, and S3 test pins. Cu was a common ingredient in all the test pins, and this was confirmed through the XRD spectrum, as shown in Figure 2. The details of the X-ray diffraction analysis for the constituent’s present are seen in Figure 2b. The radiation of the X-ray Cu-k was transmitted through the samples, and X-ray patterns were attained. From Figure 3, it can be seen that Sample S had the highest peak for aluminium. In contrast, other peaks were visible for Cu. From Sample S2, the three peaks with the highest strength were clearly apparent for aluminium, B_4_C, and Gr. For aluminium, the highest peak was clear and while several peaks were apparent for B_4_C, Gr, and Cu in Samples S3 and S4. After comparing the experimental peaks with regular peaks, various strengthening’s, such as aluminium B_4_C and Gr, were detected. The existence of B_4_C and Gr was seen by X-ray diffraction.

### 3.2. Density and Porosity

The density of the prepared AMMCs was considered through the rule of mixture and Archimedes principle for theoretical and experimental density, respectively. The changes in density values concerning the increasing strengthening particles (wt.%) are shown in Figure 3. By switching to a hybrid metal matrix composite design, the obtained AMMCs were lighter than the un-reinforced Al2219 alloy. It is understood that the observed density values of the composite often deviated from the theoretical values because of the presence of pores and vacuums. It was noticed that the density value decreased from 2.73 g/cm^3^ to 2.60 g/cm^3^ with a rise in wt.% of B_4_C and Gr. The presence of porosity in the samples was within the acceptable limit of 3–6%. Other casting defects were not taken into account during the estimation of the theoretical density. The change in porosity for the samples produced is seen in Figure 3. The existence of pores could cause physical and mechanical sample properties to be reduced. The reinforced samples had less porosity (%) than un-reinforced samples because of the more substantial matrix compaction with the reinforcement. The experimental density was determined using the mathematical equation given below.

*ρ_w_* = *m*/(*m* − *m*_1_) (1)

where *m* is the mass of the composite sample in air and *m*_1_ is the mass off the same composite sample in distilled water. *ρ_w_* is the density of the distilled water.

### 3.3. Hardness Test

**Figure 4:**
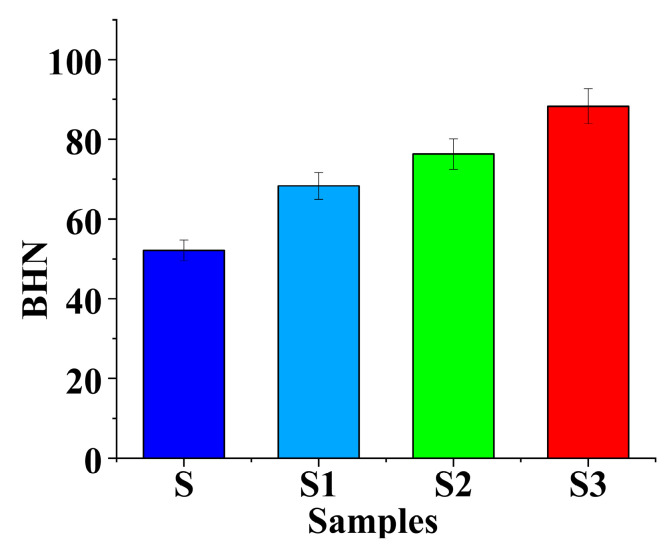
Hardness value of the samples.

It was revealed that the composite’s hardness assessed the penetration depth of the abrasive particles. Thus, an improvement in composite hardness could minimize abrasive wear by applying varying quantities of B_4_C and Gr, and the hardness of the matrix increased, as shown in Figure 4. The hardness values of the S1, S2, and S3 test samples were considerably better than for sample S. The hardness of the hybridized matrix was increased at the periphery of the particles that were distributed throughout the matrix because of a higher strain energy. The induction of a higher strength of B_4_C and Gr in the matrix provided greater resistance to penetration.

### 3.4. Wear Behavior

**Figure 5:**
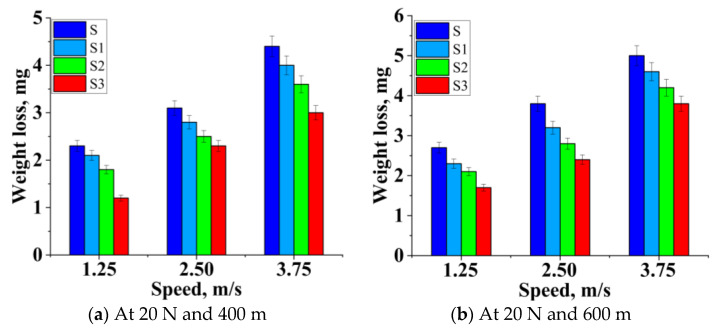

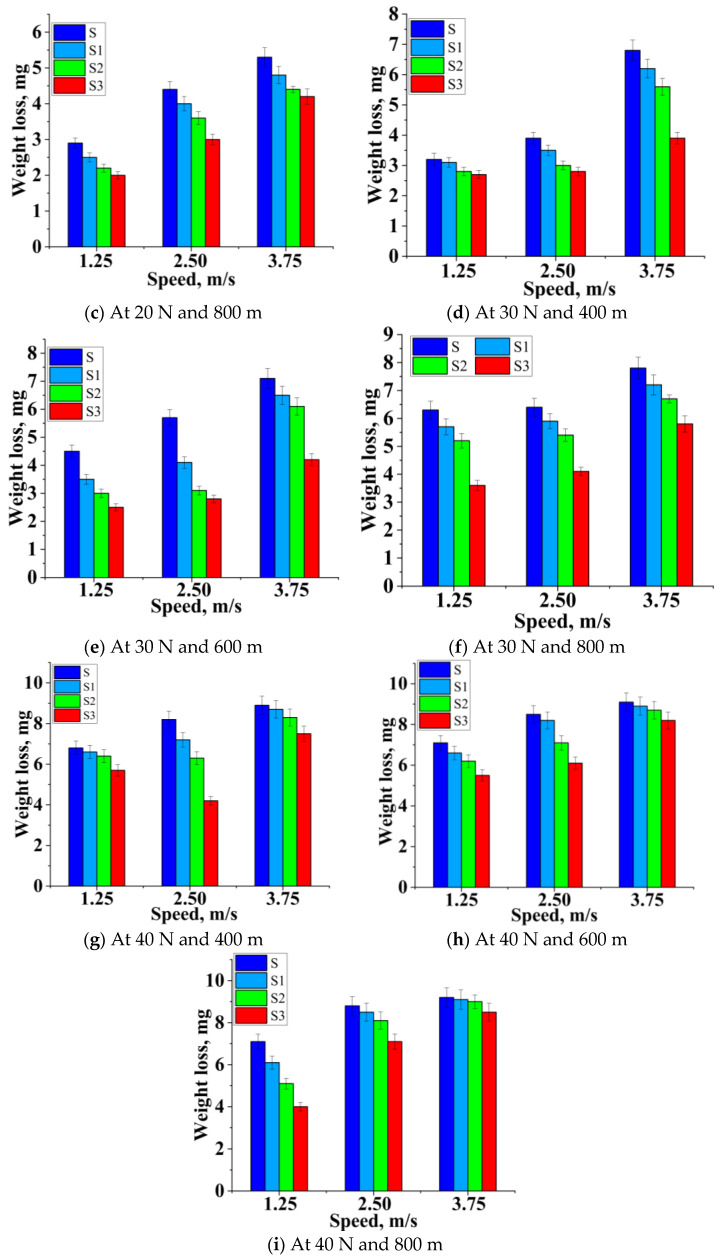
Effect of sliding velocity vs. weight loss at different load and distance (**a**–**i**).

The test sample’s weight loss was measured using a pin on a disc tribometer by varying the wt.% of B_4_C and Gr along with the variation of load, speed, and distance. Experiments were performed in compliance with the test conditions stated in the Taguchi model-L27. Sample S was less resistant to wear than Sample S1; this implies the weight loss of Sample S1 with B_4_C and Gr was less than that of graphite-free Sample S. The inclusion of B_4_C and Gr particles reduced the weight loss of the hybridized matrix. The thin layer’s deposition on the surface of Sample S1 was attributable to the decrease in weight loss, as shown in Figure 5 [45,46,47,48].

It has been noted that the rate of weight loss decreased almost linearly with the increasing strengthening’s. As Sample S exhibited a very thin layer over a period, it was diminished, and the surface was exposed to the hard surface during sliding. The stability of the layer and the detoration rate of the layer were very high; thus, the weight loss of Sample S increased. The unreinforced Sample S weight loss was very high (47% in contrast with Sample S3).

The hardness of the material was clearly linked to the improvement in wear resistance of the sample. Because of the existence of strengthening, the mechanically mixed layer was formed during sliding, but the stability of the MML layer greatly influenced the wear resistance of the hybridized sample [28].

Figure 5a–c indicates the weight loss of the composites with varying strengthening ratios compared with the sliding velocity of 1.25 m/s, 2.5 m/s, and 3.75 m/s. The findings showed that the amount of weight loss dropped significantly as the percentage reinforcement rose. The weight loss for the composites increased as the speed increased from 1.25 m/s to 3.75 m/s over the sliding distance of 400–800 m. At a higher velocity, the tribo-layer was damaged because of the removal of more particles from the matrix, which further influenced the ploughing action on the sliding surfaces. The wear resistance of the hybrid composites was improved by 40% compared with the unreinforced alloy. Reinforcement materials formed a mechanically mixed layer (MML), and the stability of this layer also depended on the interfacial bonding of the strengthening particles; hence S3 exhibited more wear resistance.

Figure 5d–f illustrates the specimens subjected to a wear test with a load of 30 N and a sliding velocity of 1.25 m/s to 3.75 m/s. The material was withdrawn from the ploughing because of the higher load and speed; thus, the mechanically mixed layer extracted and formed a mild surface over the sliding specimen. The increased load and speed induced fatigue on the composites’ surface, leading to cracks of the surface. SEM images showed that a cavity was formed by the agglomerated particle removed from the matrix, and sub-surface exposure was observed. The hybrid composite (S3) showed a better wear resistance, of about 25%, than the unreinforced alloy (S).

Figure 5g–i illustrates that with the increased velocity, there was no appreciable movement of material from the test sample; later, it began to increase because of the breakdown of the resistive lubricated tribo-layer. As soon as wear started, the strengthening particles became wear residue [26]. The wear resistance of Sample S3 was higher than for Sample S. On the other hand, it displayed the greater wear resistance at a lower speed, as shown in Figure 5g–i. The splashed Gr particles from the worn composite surface formed a thin and rich tribo layer that prevented direct metallic interaction between pin and disc. When the load was below 30 N, the reinforced composite weight loss was more minor than for the unreinforced alloy. If the load rose above 30 N with a sliding speed of 3.75 m/s, all the samples’ weight loss increased, as seen in Figure 5g–i.

At a sliding distance of 400 m, a substantial improvement in wear resistance was found [48,49,50]. Strengthening particles were withdrawn or broken down as the test sample passed across the disc. The collective flow of the material between the reinforced pin and steel disc contributed to creating a mechanically mixed layer. The mechanically mixed layer offered a hardcover, which protected the test sample from improving the wear resistance. Once wear was commenced on the surface of the sample, strengthening particles became wear debris.

**Figure 6:**
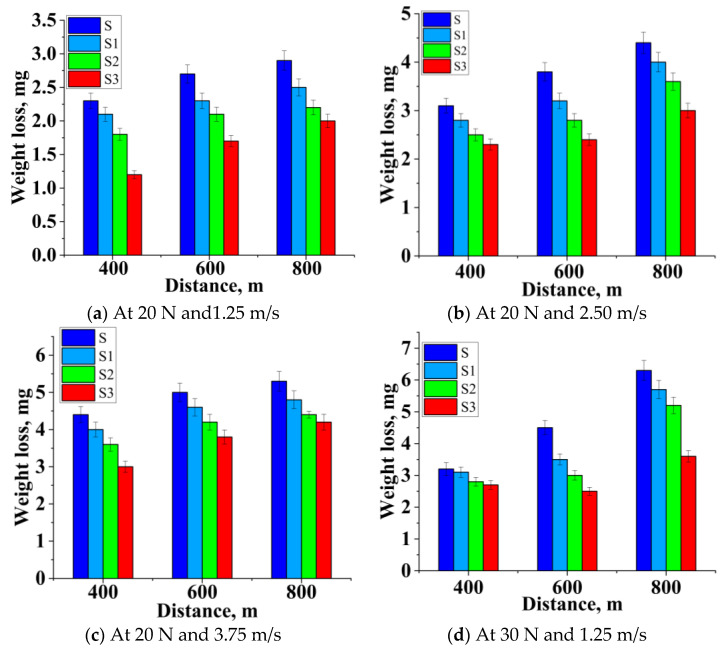

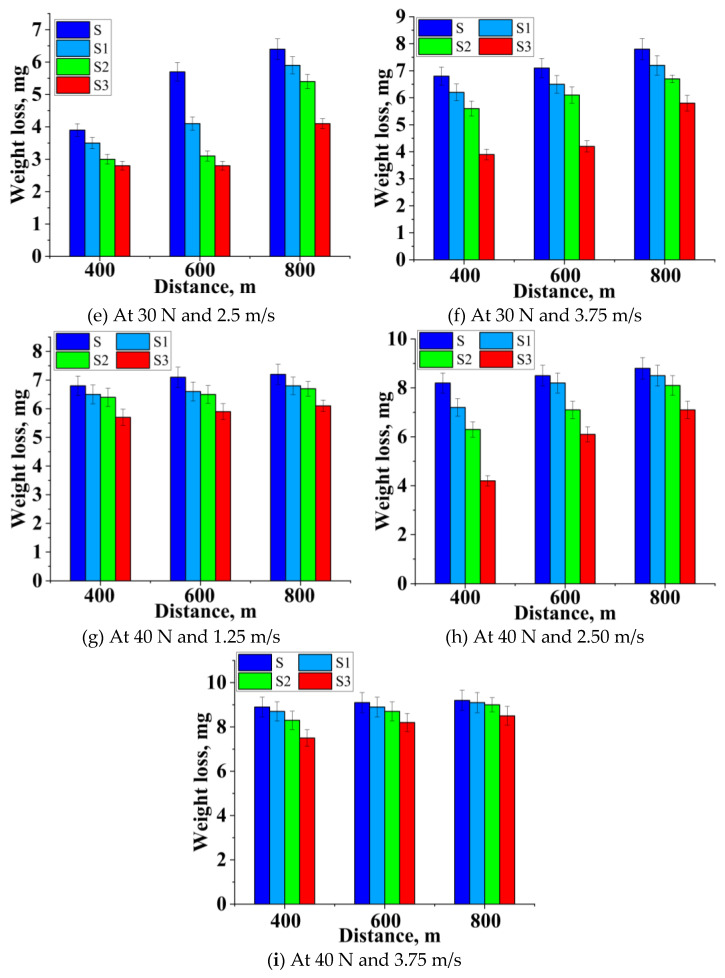
Effect of sliding distance vs. weight loss at different loads and speeds (**a**–**i**).

Debris was also a part of the tribo-layer. The virgin surface of the sample was not in contact with the disc [51]. Therefore, there was not much material movement, as shown in Figure 6. Sample S3 had less wear than the other samples due to the existence of a higher wt.% of B_4_C and Gr (15% B_4_C and 5% Gr). Strengthening particles were withdrawn or broken down as the test sample passed across the disc, contributing to the collective flow of material between the reinforced sample and steel disc to create mechanically mixed layers, as seen in Figure 6a–c. The mechanically mixed layer offered a hardcover, which would protect the sample; thus, wear resistance was improved.

Figure 6d–f indicates that as the sliding distance increased, the component’s weight loss also increased, and a similar trend was continued in all the samples. From Figure 6d–f it is seen that the reinforced alloy showed a better wear resistance because of the addition of reinforcement, and the thickness of this MML increased. This layer allowed for a small amount of new metal-to-metal interaction. The removal of this layer must be supplemented by the addition of a new material to the substrate. As a result of the applied load (30 and 40 N), extreme wear was observed. However, the hybridized composite showed a better wear resistance (11%) than Al2219. Thermal softening happened at greater loads; thus, the soft pin layer was decomposed and enabled the direct metallic interaction, leading to wear. MML protected the wearing surface. This layer avoids direct contact, which led to a reduced wear rate.

The rise in load and speed impaired the tribo-layer because of an increase in friction over the layer, which contributed to a corresponding decrease in the region protected by the layer, which caused the wear to increase. As the load and speed increased simultaneously, the weight loss of the hybrid test samples also increased. On the other hand, the Sample S3 acquired a lower weight loss in all the conditions compared with Sample S. The weight loss was mutually influenced by load and velocity. AMMCs samples displayed lower weight loss at lower loads, speeds, and distances, reducing the worn surface area because of the Gr and B_4_C particles’ presence in the MML layer. The MML layer was the product of B_4_C and Gr, and it avoided the direct metal interaction that improved wear resistance, as visible in the SEM micrograph, confirming the oxidation reaction [51,52].

**Figure 7:**
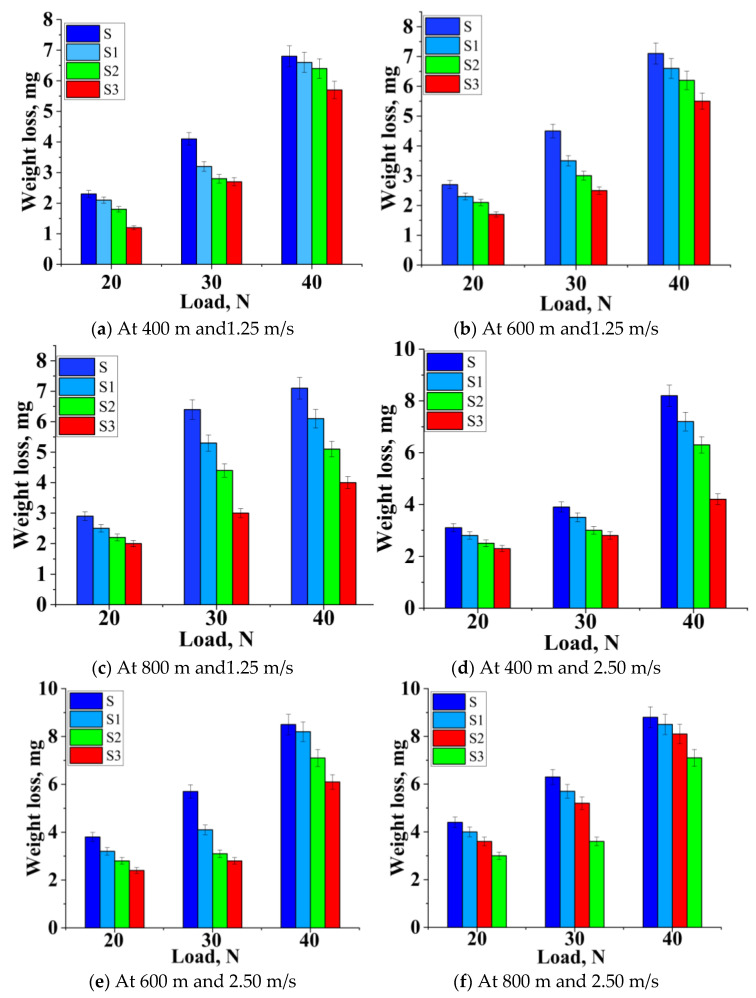

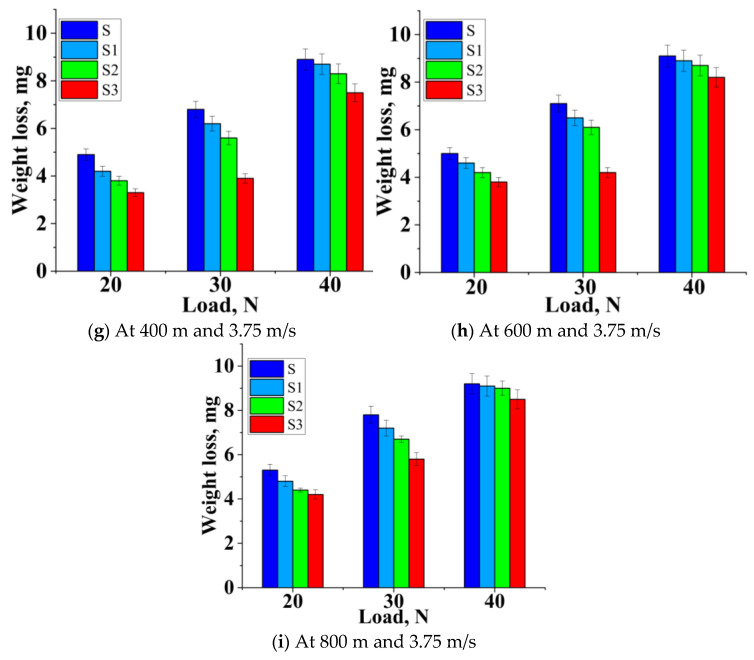
Effect of load vs. weight loss at different sliding velocities and distances (**a**–**i**).

The rise in load impaired the MML because of an increase in friction over the layer, which contributed to a corresponding decrease in the region protected by the layer, which caused the wear to increase. As the load and speed increased simultaneously, the hybridized test samples’ weight loss also increased, as seen in Figure 7a–c. On the other hand, all the reinforced samples acquired a lower weight loss at all the conditions in contrast with unreinforced alloy 2219. The weight loss depended on the load and speed. AMMC samples demonstrated a lower weight loss at a lower operating load and speed, thereby reducing the wear region because of Gr and B_4_C particles’ inclusion. The MML was generated by B_4_C and Gr. It prevented direct metal interactions that enhanced the composite’s resistance, as visible in SEM, and confirmed the oxidization reaction (oxide layer).

When the load was higher than 30 N with a sliding speed of 2.75 m/s, there was an increasing trend in the wear rates for all the samples. Figure 7d–f demonstrates that the slope between wear and strengthening (percentage) decreased with the load increasing, because of the matrix’s probability of holding reinforcement particles at a low sliding speed. As the sliding velocity increased from 1.25 m/s to 3.75 m/s, the slope between the wear rate and load was almost similar for all the percentage reinforcements until 40 N. Figure 7d–f shows the rise in weight loss as the load was higher than 30 N as a result of the delamination of plastically deformed surfaces, as seen in the SEM images.

As the load increased from 20 N to 40 N, the same phenomenon occurred at an even higher sliding distance and velocity, which could be identified from the trend shown in Figure 7g–i. When the applied load was 40 N, the weight loss of the samples followed a similar trend. Deformation of MML was common in all the samples, but more stability was possessed by the reinforced alloy than the unreinforced alloy. Marginal improvements in Sample S3 were noted (7.6%) at a higher load when compared with Sample S.

### 3.5. Worn Surface Morphology

**Figure 8:**
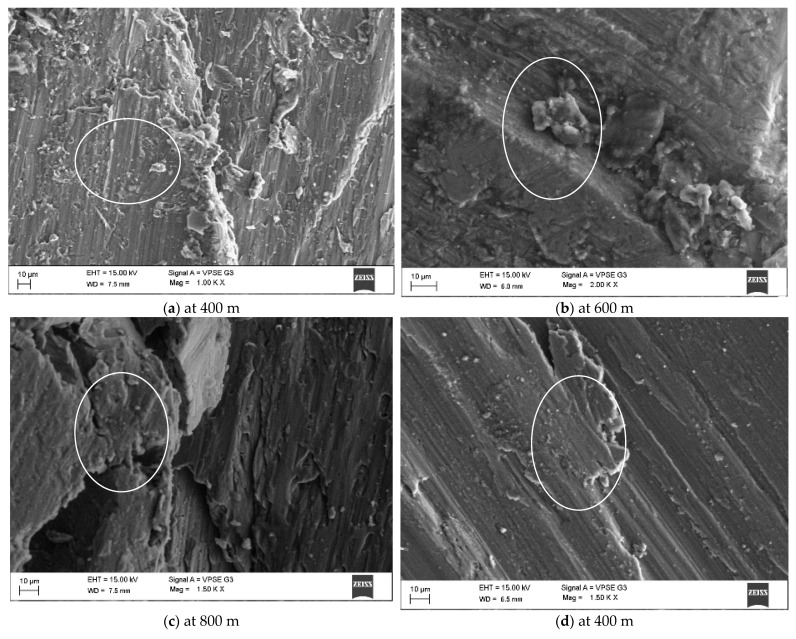

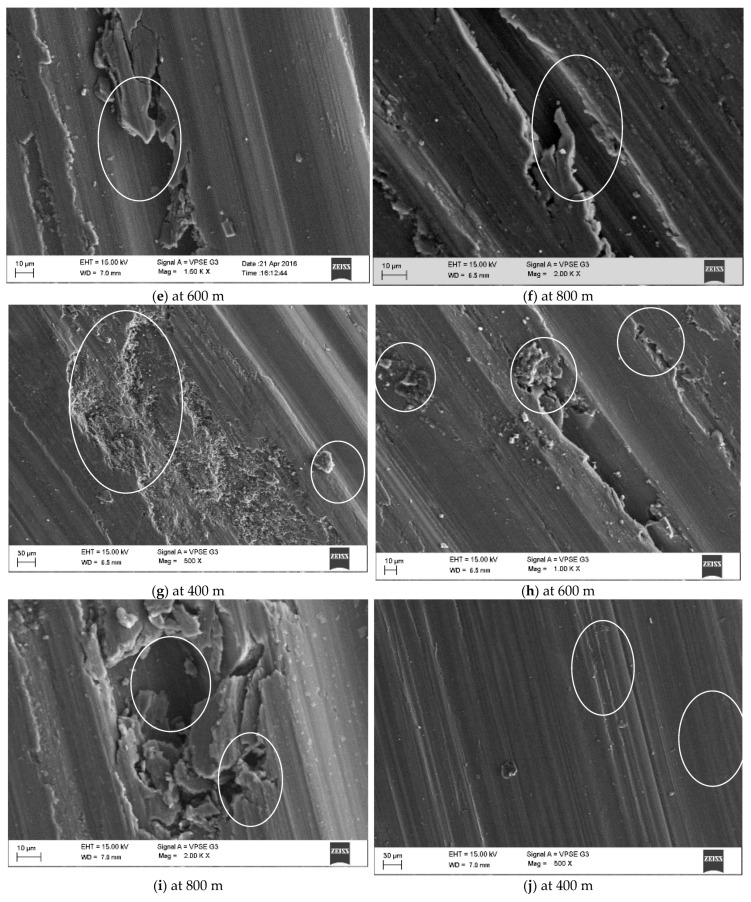

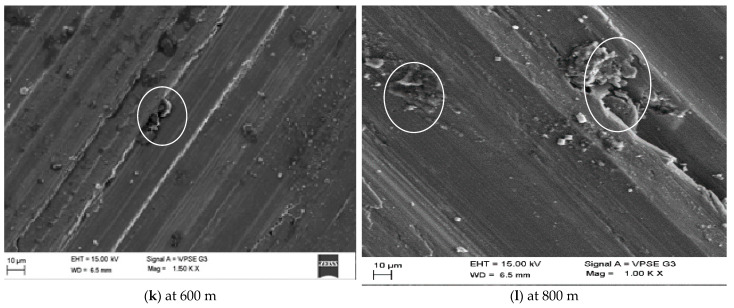
Worn surface of the samples with respect to sliding distance: (**a**–**c**) S, (**d**–**f**) S1, (**g**–**i**) S2, and (**j**–**l**) S3.

Figure 8a–c illustrates the un-reinforced alloy’s SEM micrographs, and the worn surface was parallel to the sliding direction. Al2219 alloy’s (Sample S) worn surface revealed a re-melt bubble and high flow of materials with little cavity, clearly showing the adhesive wear mechanism. As seen in Figure 8a, at a higher magnification, scratches and cutting were noticed with detached wear debris, forming deeper grooves in the direction of sliding. Wear arose through the delamination process, as the strengthening particles were stripped from the surface, as seen in Figure 8d–f. In addition, for Sample S1, the SEM micrograph displayed long deformation bands, a strong sign of delamination wear [35]. The surface of Sample S1 revealed deeper grooves and less plastic flow, which are direct signs of improved wear resistance, as seen in Figure 8g–i. The lubrication layer collapsed and induced a higher degree of weight loss, which coincided with the experimental results. B_4_C and Gr in Sample S1 exhibited a rich tribo layer that avoided direct metal to metal contact. The tribo-layer on the surface of Sample S1 was recommended as the defensive layer and showed a decrease in weight loss [45]. The interfacial strength of Al2219 and B_4_C and Gr also influenced the wear behaviour. The higher interfacial bond strength could result in better resistance to abrasion [43], as shown in Figure 8g–i. Sample S2 exhibited less weight loss than Samples S and S1 because of the existence of a strong tribo-layer, and this tribo-film stopped penetration from wear debris, which led to less damage to the surface, as shown in the SEM micrograph. Strengthening particles were withdrawn or broken down as the test sample passed across the disc; this contributed to the collective flow of materials between the reinforced sample and steel disc to create mechanically mixed layers, as seen in Figure 8g–l. The mechanically mixed layer offered the hardcover to protect the test sample, so that the wear resistance was improved. Hence, the worn surface of Sample S3 showed smooth patches and small scratches. When considered with all the Samples, from S to S3, Sample S3 had a thick mechanical mixed layer acting as lamination, which prevented the surface damage and maintained a good hardness (88.3 BHN).

### 3.6. Response Surface Analysis

RSM is an efficient analytical approach for experimental design. Furthermore, RSM can describe multiple variables with the minimum resources, quantitative details, and appropriate test design on the several variables and factors affecting a response variable at all times. RSM was used to describe the parameter consequence, and it also included the interaction between the defined parameters. The RSM study was used to examine the effect of the wear parameters on the samples’ weight loss. These criteria have been set at various levels, such as three levels for load, speed, and distance, based on the combination of all the parameters, and their level mathematical model was formed. This helped to describe the effect of the parameters. The experimental method had 2400 cycles and was investigated after that; experiments were randomized. RSM described each variable based on the key influence and interaction effects on the output at each step [53]. The multivariate model is given as follows:

Y = β_0_ + ∑^3^_i=1_ β_i_ X_i_ +∑^3^_i=1_ β_ij_ X_i_X_j_ +∑^3^_i=1_ β_ijk_ X_i_X_j_X_k_ (2)

Design expert-17 was used to run a regression operation on a quadratic equation for mathematical analysis and to determine which variables were influenced by the coefficients and parameters.

**Figure 9:**
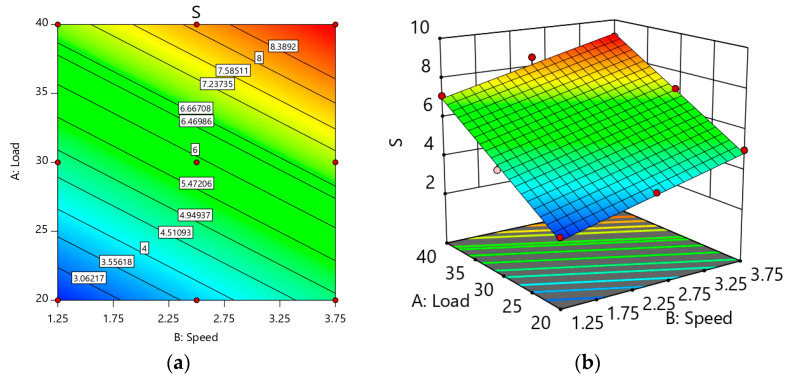
(**a**) 2D Response surface plot effect of load and speed on the weight loss of Sample S; (**b**) 3D Response surface plot effect of load and speed on the weight loss of Sample S.

Figure 9 depicts the influence of load and speed on Sample S as a response surface map. The weight loss variation as far as the association of parameters can be understood thoroughly from these graphs. The optimum weight loss was achieved at 40 N load and 3.75 m/s speed (Figure 9a,b). The material was withdrawn from the ploughing because of the higher load and speed; thus, the mechanically mixed layer was extracted and formed a mild surface over the sliding specimen. The increased load and speed induced fatigue on the sample (S) surface, leading to cracks. The load at 20 N weight loss was less when the load increased to 3.75 N; 62% of weight loss increased (Figure 9b). When the speed increased to 3.75 m/s, the weight loss increased, but it was less than the weight loss obtained at 40 N. This is because the interfacial pressure between the sample and the disc surface varied with the load.

**Figure 10:**
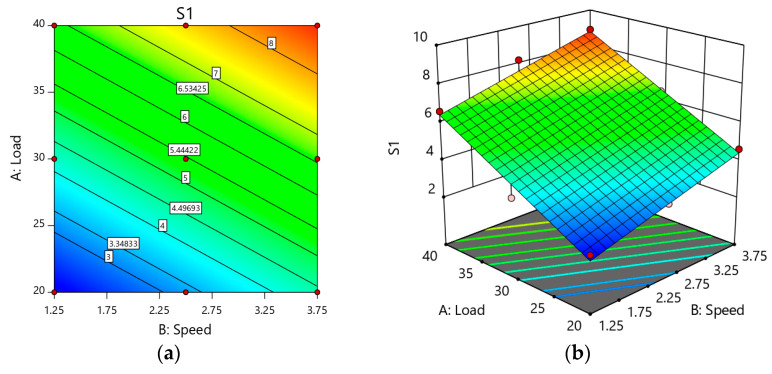
(**a**) 2D Response surface plot effect of load and speed on the weight loss of Sample S; (**b**) 3D Response surface plot effect of load and speed on the weight loss of Sample S1.

A similar trend was seen in the weight loss result of Sample S1, as illustrated in Figure 10. The contact pressure was less at a low load of 10 N, leading to a lower weight loss of the sample and surface damage. Concerning the applied load, contact pressures appeared to increase linearly. As the load was maximized (40 N), sample deformation could lead to increased material removal and had implications for the sample’s high weight loss. The RSM plots showed that the weight loss of Sample S2 initially decreased and then increased as the sliding speed increased (3.75 m/s) as far as both loads were concerned. The material was discarded as residue that may have lodged between the disc and sample surfaces, leading to more significant weight loss. When the load was higher than 30 N with a sliding speed of 2.75 m/s, there was a growing wear rate for all the samples. Figure 10b demonstrates that the slope between wear and strengthening (percentage) decreased with the load increasing because of the matrix’s probability of holding reinforcement particles at a low sliding speed. As the sliding velocity increased from 1.25 m/s to 3.75 m/s, the slope between the wear rate and load was almost similar for all the percentage reinforcements until 40 N. Figure 10a–b shows the rise in weight loss as the load was higher than 30 N as a result of the delamination of plastically deformed surfaces, as seen in the SEM images.

**Figure 11:**
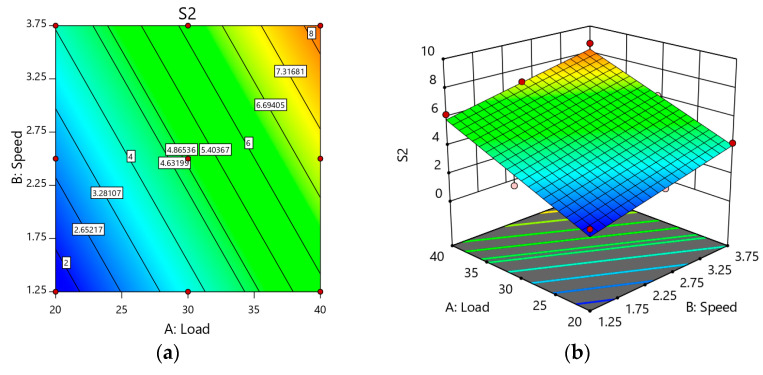
(**a**) 2D Response surface plot effect of load and speed on the weight loss of Sample S; (**b**) 3D Response surface plot effect of load and speed on the weight loss of Sample S2.

When the speed was lower (1.25 m/s), the contact time between the disc and sample surface was less likely to allow for less metal contact and a higher degree of weight loss. When the speed approached 3.75 m/s, a high interface temperature was produced, and the material was oxidized. These findings are anticipated to produce an oxide coating known as an MML on the surface of the specimen. Thus, weight loss was reduced. Weight loss was increased when speed rose from 1.25 m/s to 3.75 m/s; the same trend was observed in Figure 11a. This may be caused by the failure of an un-stable MML layer. The rise in load impaired the MML because of an increase in friction over the layer, which contributed to a corresponding decrease in the region protected by the layer, which caused wear to increase. As the load and speed increased simultaneously, the hybridized test samples’ weight loss also increased, as seen in Figure 11a, b. On the other hand, all the reinforced sample acquired a lower weight loss at all the condition compared with unreinforced alloy 2219. The weight loss depended on the load and speed.

**Figure 12:**
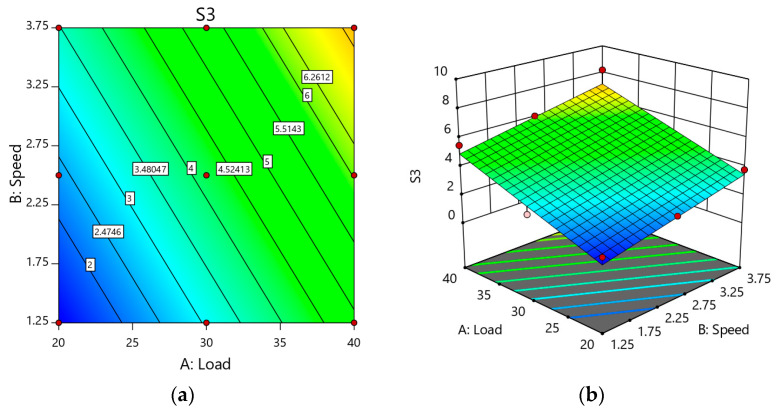
(**a**) 2D Response surface plot effect of load and speed on the weight loss of Sample S. (**b**) 3D Response surface plot effect of load and speed on the weight loss of Sample S3.

Figure 12 illustrates the RSM plot of Sample S3. A similar trend was seen as the load increased from 20 N to 40 N, where the weight loss increased, but it was less compared with the other samples. When the applied load was 40 N, the weight loss of the samples followed a similar trend. Deformation of MML was common in all the samples, but more stability was possessed by the reinforced alloy than the unreinforced alloy. Marginal improvements of Sample S3 were noted (7.6%) at a higher load when compared with Sample S. Speed also reduced the stability of the MML, so that weight loss increased as speed and load increased.

### 3.7. ANN (Artificial Neural Networks) Analysis

ANN requires complete results of the experiment, along with the variables and feed through the input nodes. The output nodes were used to generate output values, which were then compared with the actual values. The processes were done through backpropagation neural networks, and errors could be calculated. Normalized root mean square was used to check and evaluate the performance of the ANN [33]. Three inputs (L: load, S: speed, D: distance) were used to construct the ANN architecture for all the samples in which weight loss (w) was the output, and the hidden layer indicated that an interaction between the neurons was not visible [54,55,56,57].

#### 3.7.1. ANN Analysis for Sample S

**Figure 13:**
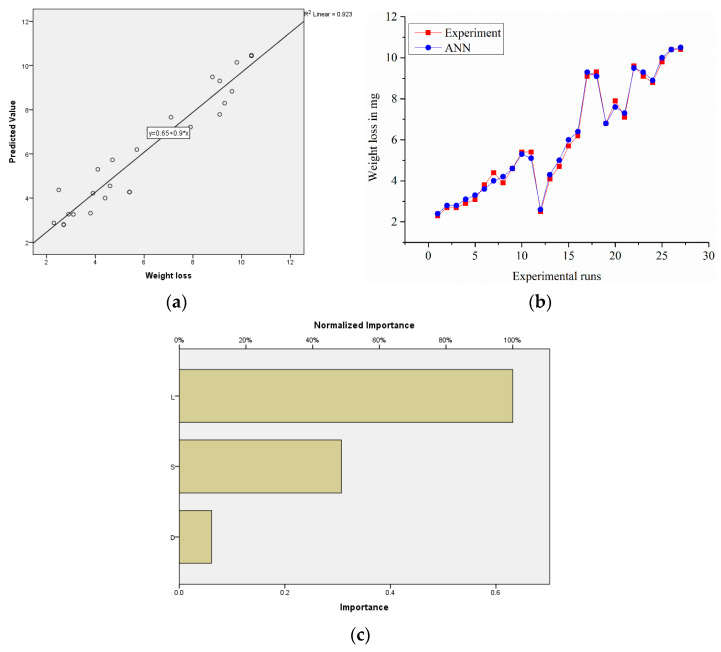
(**a**) Experimental vs. predicted values of the samples; (**b**) error-values; (**c**) normalized factor importance.

A total of 27 experimental data points were used to create a fully developed feedback propagation network. Sigmoid logistic was used to activate the hidden layer. The training was carried for 100,000 cycles, and further iterations were not favourable. This test was used to examine the architecture. The experimental values were compared with ANN’s expected values in order to verify the network’s generalization efficiency. The neural network created adequate agreement between the experimental results and ANN results, as seen in Figure 13a. The relative error was 0.04, R^2^ = 0.92, and it was confirmed that the load and speed dominated the increase in weight loss, as seen in Figure 13c.

#### 3.7.2. ANN Analysis for Sample S1

**Figure 14:**
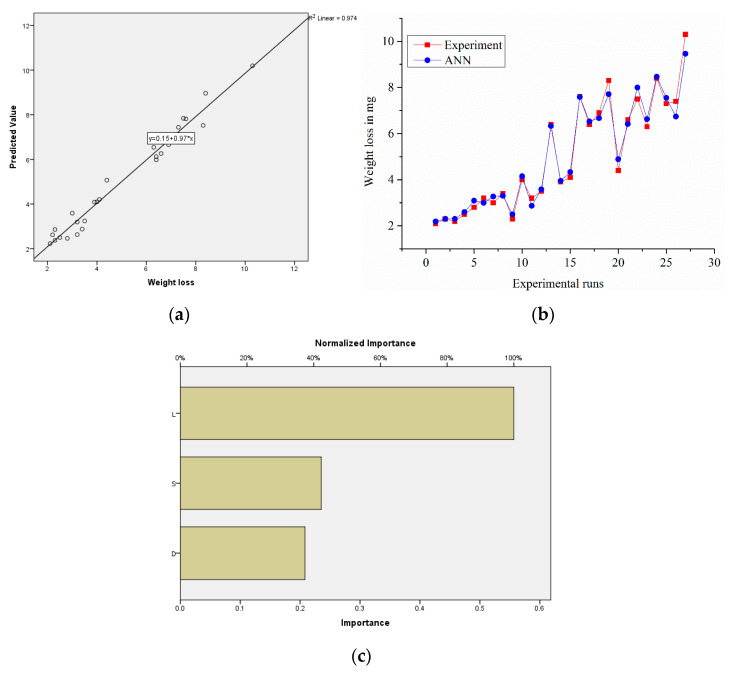
(**a**) Experimental vs. predicted values of the samples; (**b**) error-values; (**c**) normalized factor importance.

The IBM SPSS Statistics-22 split the input vectors and target vectors randomly into two sets: 80% used for training and 20% for validating the system. The correlation coefficient for weight loss of 0.974 between the expected and experimental values was calculated using the whole dataset. This is a clear indicator that the model was correct. An ideal prediction would have all the points on the central axis; each approach’s precision could easily be compared by the proximity of the data groups in this line. A diagonal line showed a better linear fit. Figure 14a shows that several of the values came closest to the central line. The absolute relative error was 0.057, and this value confirmed the accuracy of the prediction method. These error levels were acceptable and lower than the usual errors caused by experimental variance and instrumentation precision, as seen in Figure 14b.

#### 3.7.3. ANN Analysis for Sample S2

**Figure 15:**
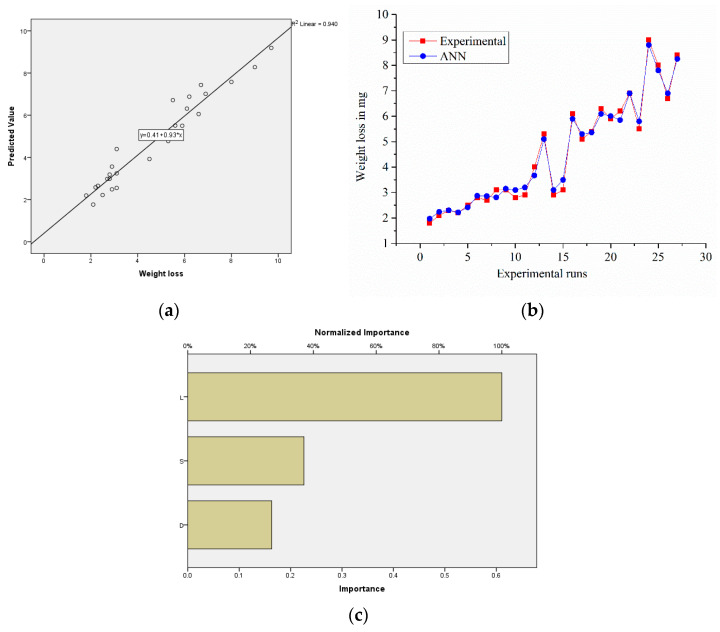
(**a**) Experimental vs. predicted values of the samples; (**b**) error-values; (**c**) normalized factor importance.

The relative error from the experimental weight loss was 0.028%. As a consequence, variations in expected values were closer to the experimental values, as shown in Figure 15b. Normalized importance graphs (Figure 15c) revealed that the load more effectively raised the weight loss for Sample S2. Figure 15a reveals points scattered along the diagonal line; this kind of scattering is a good sign for accuracy in the prediction of ANN [33,34]. The results show that, for all the input parameters, the neural network projections agreed very well with the experimental values (R^2^: 0.940). The estimated (ANN) and experimental values were found to be consistent with the similar weight loss patterns for the conditions examined.

#### 3.7.4. ANN Analysis for Sample S3

**Figure 16:**
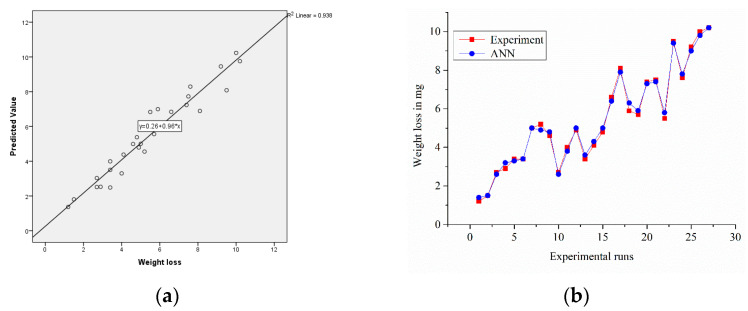

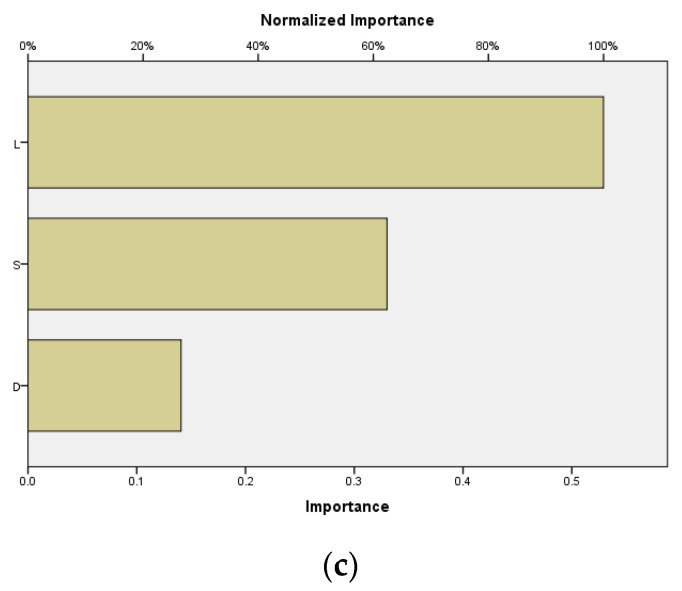
(**a**) Experimental vs. predicted values of the samples; (**b**) error-values; (**c**) normalized factor importance.

A model for the artificial neural network was developed to predict the weight loss of composite materials of Al2219 + B_4_C − Gr, including the impact of operating conditions. An adequately qualified neural network was predicted based on test conditions. In the mean error range of 0.029%, the network estimated the weight loss. The performance parameters for any intermediate inputs could also be achieved using qualified ANN values. This value confirmed the accuracy of the prediction method. These error levels were acceptable and lower than the usual errors caused by experimental variance and instrumentation precision, as seen in Figure 16b. Figure 16a reveals points scattered along the diagonal line; this kind of scattering is a good sign for accuracy (R^2^: 0.938) in the prediction of ANN [33,34]. Normalized importance graphs reveal that the load more effectively raised the weight loss for Sample S3, as seen in Figure 16c.

## 4. Conclusions

- Hybridized composites were successfully fabricated by the stir-casting process.
- The reinforced matrix was harder (37%) than the base alloy because of the increased ceramic phase in the Al2219 matrix.
- The inclusion of the B_4_C and Gr particles served as a barrier to dislocation and contributed to a greater hardness than the Al2219 matrix.
- It was found that hardness was directly proportional to wear resistance.
- The inclusion of B_4_C and Gr particles could be attributed to the induction of a greater strength to the matrix by providing greater wear resistance.
- The weight loss was significantly less for the 15 wt.% B_4_C and 5 wt.% Gr, because of the existence of a mechanically mixed layer that contained B_4_C and Gr.
- The weight loss of Sample S3 (47%) was significantly less than for Sample S.
- ANN results confirmed that the load and speed contributed to the weight loss of the composites.

## Figures and Tables

**Table 1:** Constituents of Al2219 [37].

Constituents	Si	Mg	Zr	Cu	Fe	Mn	Ti	V	Zn
Weight %	0.2	0.02	0.1–0.25	5.8–6.8	0.3	0.02	0.02	0.05	0.1

**Table 2:** Percentage of particulate reinforcement by weight [36].

Sample Designations	Composition	Al2219, %	B_4_C, %	Gr, %	Total Particulate B_4_C and Gr Reinforcement, %
S	Al 2219	100	0	0	0
S1	Al2219 + 5% B_4_C − 5% Gr	90	5	5	10
S2	Al2219 + 10% B_4_C − 5% Gr	85	10	5	15
S3	Al2219 + 15% B_4_C − 5% Gr	80	15	5	20

## Data Availability

Raw data available from the corresponding authors upon request.
